# Unsolved Issues in Anhedonia: A Call for Targeted Inquiry

**DOI:** 10.62641/aep.v53i5.1993

**Published:** 2025-10-05

**Authors:** Maria Luca, Antonina Luca, Alessandro Serretti

**Affiliations:** ^1^Centre for Addiction Adrano-Bronte, 95031 Adrano, Italy; ^2^Department of Medicine and Surgery, Kore University of Enna, 94100 Enna, Italy; ^3^OASI Research Institute – IRCCS, 94018 Troina, Italy

Anhedonia, defined as the reduced ability to experience pleasure or interest in activities that are typically rewarding, remains one of the most enigmatic and clinically challenging symptoms of major depressive disorder (MDD) and is often used to diagnose it [1].

Despite its inclusion as a core criterion in the DSM-5 diagnostic framework for MDD, anhedonia is increasingly recognized as a transdiagnostic construct of several psychiatric (e.g., autism spectrum disorder, attention-deficit hyperactivity disorder) and neurological (e.g., Parkinson’s disease and vascular dementia) conditions [2, 3].

From a clinical point of view, two facets of anhedonia can be recognized, namely anticipatory (reduced ability to experience pleasure in anticipation of rewarding stimuli) and consummatory (reduced ability to experience pleasure from rewarding stimuli) [1].

Contemporary models frame anhedonia as a disruption in the reward processing system [1]. From a theoretical point of view, the reward system encompasses two main components, namely *liking* (hedonic impact: derive pleasure from in-the-moment experiences) and *wanting *(motivational salience: incentive value for pursuing a reward, closely associated with anticipatory pleasure), along with *learning* (acquisition of reward-related associations, representations and predictions: encompassing both anticipatory and consummatory components) [4]. Building upon this model, current research associates consummatory anhedonia to deficits pertaining *liking*, while anticipatory anhedonia is typically re-conducted to altered *wanting* [5]. However, the domains of anhedonia may be even more complex than this, and further refinements may arise. For example, the term decisional anhedonia has been introduced to highlight the impact of anhedonic features on *choosing* behaviours as well. Considering that the components of the reward system are regulated by interrelated, yet distinct, neurobiological mechanisms, what might seem a theoretical concern acquire a strong clinical value, since the different domains of anhedonia may show different responses to treatment. Indeed, the historically recognized central role of hypodopaminergic functioning in the anhedonic experience may be rather marginal in relation to consummatory anhedonia, being more crucially involved in the motivational aspects of reward of the mesolimbic pathway [6]. Overall, several dopaminergic and non-dopaminergic circuits, such as mu-opioid and endocannabinoid signalling, along with altered processing among brain structures (e.g., orbitofrontal and anterior cingulate cortex) may contribute to the complex phenomenon of anhedonia (or, more precisely, anhedonia*s*). Further pathophysiological mechanisms may include inflammation and immune-metabolic factors such as IL-6, TNF-alpha and CRP that have been shown to predict blunted ventral-striatal responses and motivational deficits in patients, suggesting that cytokine modulation could normalise reward signals opening the research hypothesis of biomarker-guided anti-inflammatory or dopaminergic therapy [7, 8]. Sex specific approaches should also be considered [9].

However, despite this refined theoretical understanding, the rating scales for depression of frequent clinical use do not distinguish between anticipatory and consummatory pleasure, failing to capture the complexity of anhedonia. This rather generic approach may explain why research on the topic (from neurobiological underpinnings to therapeutic aspects) has remained elusive for years, not succeeding in bringing the desired insights. A broader use of more specific tools such as the Dimensional Anhedonia Rating Scale (DARS), that differentiates anhedonia facets such as interest, motivation and reward, could improve the resolution of future studies [10].

Another relevant challenge is the difficulty to separate anhedonia from apathy, avolition and alexithymia. Distinguishing these remains hard, especially in neurodegenerative disorders, it is therefore important to use a specific set of tools investigating the different dimensions in order to correctly dissect the whole clinical presentation.

In this convoluted landscape, one of the most pressing clinical challenges is the relative resistance of anhedonia to first-line pharmacological treatments. Novel therapeutic strategies (e.g., ketamine, repetitive transcranial magnetic stimulation, psilocybin) have shown preliminary promise [11, 12]. However, evidence remains limited and caution must be applied, also in relation to the accurate selection of patients who may benefit from these options.

Another unsolved issue pertains to the temporal trajectory of anhedonia in MDD. As a matter of fact, increasing evidence suggests that anhedonia not only may predict non-response to treatment, but it may also constitute a residual symptom or an unwanted effect of antidepressant treatments [13, 14, 15]. Awareness of those possibilities could improve both clinical routine and research studies.

Moreover, the importance of anhedonia is not confined to MDD. Indeed, its transdiagnostic nature raises the question of whether anhedonia reflects a shared neurobiological endophenotype or whether it arises from distinct mechanisms across disorders.

**Fig. 1.**
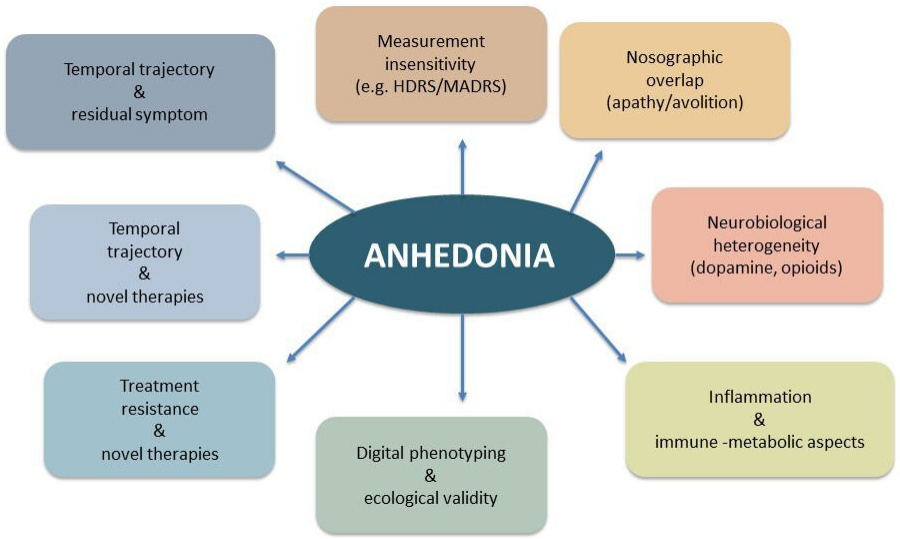
**Anhedonia in focus**. The Figure summarizes the complex and/or unresolved issues pertaining anhedonia. HDRS, Hamilton Depression Rating Scale; MADRS, Montgomery-Äsberg Depression Rating Scale.

In conclusion, anhedonia remains a deeply complex and multifaceted dimension posing significant theoretical, diagnostic, and therapeutic challenges (see Fig. 1). Its multidimensional and transdiagnostic nature requires a more multifaceted approach to both measurement and treatment, informed by advances in neuroscience and precision psychiatry. Suggestions include adopting phase-specific, psychometrically robust comprehensive assessments; integrating biomarkers including immune, metabolic and circuit-level signatures to guide possible mechanism-matched interventions; and leveraging digital phenotyping for real-world monitoring to increase ecological validity. Addressing these unsolved aspects is not only a matter of scientific curiosity, but rather an ethical imperative to improve treatment outcomes.
